# Role of creatine shuttle in colorectal cancer cells

**DOI:** 10.18632/oncotarget.28436

**Published:** 2023-05-19

**Authors:** Mayu Kita, Rina Fujiwara-Tani, Shingo Kishi, Shiori Mori, Hitoshi Ohmori, Chie Nakashima, Kei Goto, Takamitsu Sasaki, Kiyomu Fujii, Isao Kawahara, Ujjal Kumar Bhawal, Yi Luo, Hiroki Kuniyasu

**Affiliations:** ^1^Department of Molecular Pathology, Nara Medical University, Kashihara, Nara 634-8521, Japan; ^2^Department of Pharmacology, Saveetha Dental College, Saveetha Institute of Medical and Technical Sciences, Chennai 600077, India; ^3^Jiangsu Key Laboratory of Neuroregeneration, Nantong University, Nantong, Jiangsu 226001, China

**Keywords:** creatine kinase B, mitochondrial creatine kinase, ATP metabolism, phosphorylation signal, stemness

## Abstract

The creatine shuttle translocates the energy generated by oxidative phosphorylation to the cytoplasm via mitochondrial creatine kinase (MTCK) and creatine kinase B (CKB) in the cytoplasm. It is not apparent how the creatine shuttle is related to cancer. Here, we analyzed the expression and function of CKB and MTCK in colorectal cancer (CRC) and investigated the role of the creatine shuttle in CRC. Compared with normal mucosa, 184 CRC tissues had higher levels of CKB and MTCK, and these levels were associated with histological grade, tumor invasion, and distant metastasis. CK inhibitor dinitrofluorobenzene (DNFB) on CRC cell lines HT29 and CT26 inhibited cell proliferation and stemness to less than 2/3 and 1/20 of their control levels, respectively. In this treatment, the production of reactive oxygen species increased, mitochondrial respiration decreased, and mitochondrial volume and membrane potential decreased. In a syngeneic BALB/c mouse model using CT26 cells pretreated with DNFB, peritoneal metastasis was suppressed to 70%. Phosphorylation of EGFR, AKT, and ERK1/2 was inhibited in DNFB-treated tumors. High ATP concentrations prevented EGFR phosphorylation in HT29 cells following DNFB treatment, CKB or MTCK knockdown, and cyclocreatine administration. Despite not being immunoprecipitated, CKB and EGFR were brought closer together by EGF stimulation. These findings imply that blocking the creatine shuttle decreases the energy supply, suppresses oxidative phosphorylation, and blocks ATP delivery to phosphorylation signals, preventing signal transduction. These findings highlight the critical role of the creatine shuttle in cancer cells and suggest a potential new cancer treatment target.

## INTRODUCTION

In the United States, colorectal cancer (CRC) ranks third in terms of both new cases and cancer fatalities at 7.9% and 8.7%, respectively [1] According to the National Cancer Research Center, CRC is the leading cause of morbidity and the second leading cause of cancer fatalities in Japan [2]. Energy metabolism is reprogrammed from oxidative phosphorylation to glycolysis in CRC because of mitochondrial morphological and functional damage at the premalignant adenoma stage [3]. In contrast, oxidative phosphorylation is known to be responsible for energy metabolism in cancer stem cells, which are responsible for metastasis and drug resistance [4, 5]. One such system responsible for the diverse roles of energy metabolism in cancer is the creatine shuttle.

After oxidative phosphorylation and glycolysis, the creatine shuttle is regarded as a third energy production mechanism. Normal tissues contain a rapidly available temporal energy buffer, a spatial energy buffer that connects adenosine triphosphate (ATP) generation sites (such as glycolysis and mitochondrial oxidative phosphorylation) and intracellular ATP usage sites (such as ATPase), and an intracellular energy buffer. These buffers have been demonstrated to function as a metabolic regulator and energy transport system [6]. The creatine shuttle is a system in which phosphocreatine (pCr), a high-energy molecule produced by phosphorylation of creatine (Cr) by mitochondrial CK (MTCK) in the mitochondria, is transferred out of the mitochondria and ATP is extracted from pCr by CKB in the cytoplasm. The creatine shuttle is highlighted in cancer as a source of energy for cancer cells that display aggressive proliferation, and aberrant creatine kinase (CK) levels are known to be associated with many malignancies and mitotic control [7]. Stability of MTCK by HER2 boosts energy supply and promotes cell proliferation in breast cancer [8]. In contrast to normal muscular tissues, sarcomas have a reduced creatine shuttle [9]. There are two isoforms of MTCK, MTCK1 and MTCK2, which differ in their tissue distribution, expression levels, and kinetic properties [10]. These MTCKs have different kinetic properties, with MTCK1 having a higher affinity for creatine and ATP than MtCK2. MtCK1 is more efficient in transferring high-energy phosphate groups between ATP and creatine at low concentrations. In this study, MTCK1 was examined.

The Human Protein Atlas shows that colorectal cancer is a cancer with high expression of both CKB and MTCK [10, 11]. The creatine shuttle is also known to be essential for the development of CRC, and liver metastasis is associated with elevated creatine kinase B (CKB) expression [10]. Although the role of the creatine shuttle in cancer is still controversial, one interpretation is that changes in the creatine shuttle are reprogramming the energy transfer system [12].

Recently, scientists have been seeking to use energy metabolism as a novel target for cancer treatment [13]; however, this goal requires a more thorough understanding of the creatine shuttle. We hypothesized that the creatine shuttle is involved in energy metabolism and other ATP supply in cancer cells. In the current study, the role of the creatine shuttle in CRC was analyzed along with its potential as a therapeutic target.

## RESULTS

### Expression of CKB and MTCK in CRC

**Figure 1:**
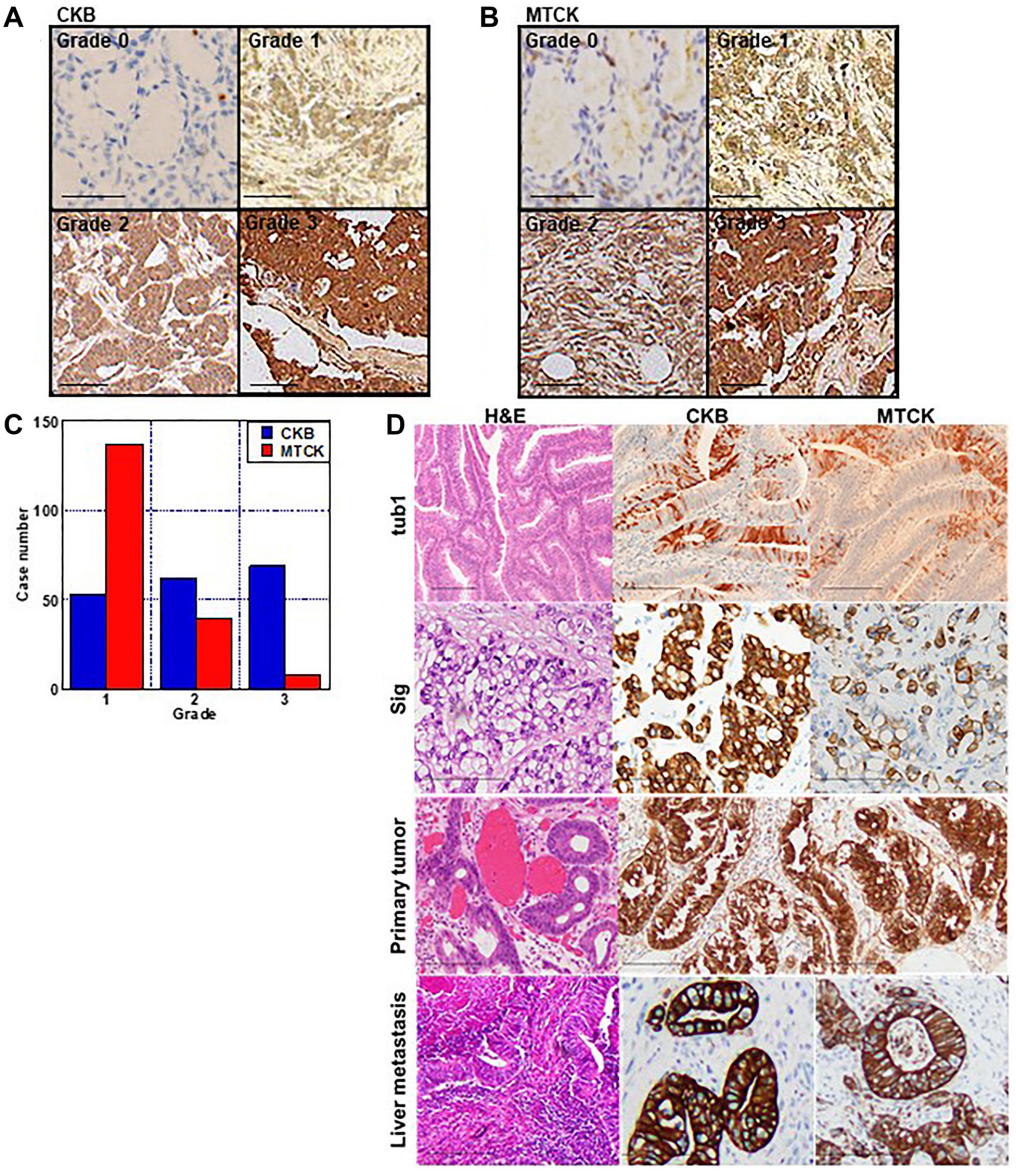
Immunohistochemistry of CKB and MTCK in CRCs. (**A**, **B**) Immunohistochemical examination of CKB (A) and MTCK (B). Immunohistochemical activity is classified into grade 0–3. Grade 0 is equivalent to the expression in the normal colonic epithelium. (**C**) Histogram of expression grade. (**D**) Immunohistochemical examination of CKB and MTCK in case of well-differentiated adenocarcinoma (tub1), signet ring cell carcinoma (sig), primary tumor and liver metastasis of tub1 case. Scale bar, 100 μm. Abbreviations: CKB: creatine kinase B; MTCK: mitochondrial creatine kinase; CRC: colorectal cancer.

We examined the expression of CKB and MTCK in CRC tissues by using tissue arrays (Figure 1). Both CKB and MTCK showed cytoplasmic immunoreactivity and were classified into grades 0–3, according to their expression levels (Grade 0 corresponds to expression in normal colonic epithelium) (Figure 1A, 1B). In CKB, the ratio of each expression grade was almost the same, whereas in MTCK, there were more cases with lower expression than in CKB (Figure 1C). Immunostaining results were compared with clinicopathological factors (Table 1). Expression of both CKB and MTCK was higher in high-grade tumors and in distant metastasis-positive cases (Figure 1D). In addition, CKB expression was higher in patients with advanced invasion (pT). When comparing expression of CKB and MTCK between primary tumor and the liver metastasis, their expression was upregulated in the metastatic foci (Figure 1D and Table 1).

**Table 1:** Expression of MTCK and CKB in 184 CRCs

Parameter^1^	Classification	*n*	MtCK^2^	*P* value^3^	CKB2	*P* value^3^
Sex	Male	110	2.1 ± 0.68	NS	1.3 ± 0.71	NS
	Female	74	2.1 ± 0.73		1.3 ± 0.70	
Age	−50 y	62	2.0 ± 0.74	NS	1.3 ± 0.72	NS
	51- y	122	2.1 ± 0.68		1.3 ± 0.69	
Histological differentiation	pap, tub1	33	1.8 ± 0.74	0.0184	1.1 ± 0.62	<0.0001
	tub2	101	1.8 ± 0.66		1.2 ± 0.71	
	por1, por2	39	2.0 ± 0.58		1.8 ± 0.68	
	muc, sig	11	2.5 ± 0.62		1.9 ± 0.63	
Histological grade	G1	33	1.8 ± 0.74	0.0014	1.10 ± 0.62	0.0303
	G2	101	1.8 ± 0.66		1.2 ± 0.71	
	G3	50	2.2 ± 0.68		1.5 ± 0.54	
Tumor invasion	pT1-pT2	20	1.8 ± 0.64	NS	1.2 ± 0.71	0.0063
	pT3-pT4	164	1.9 ± 0.68		1.7 ± 0.65	
Nodal metastasis	pN0	148	1.9 ± 0.68	NS	1.4 ± 0.68	NS
	pN1-pN2	21	2.1 ± 0.79		1.3 ± 0.72	
Distant metastasis	pM0	177	1.9 ± 0.49	NS	1.2 ± 0.71	NS
	pM1	7	2.00 ± 0.67		1.4 ± 0.64	
Primary tumor		5	2.1 ± 0.12	0.0021^4^	1.4 ± 0.13	0.0046^4^
The liver metastasis		5	2.6 ± 0.15		1.6 ± 0.2	

^1^Clinicopathological parameters were according to TNM classification [14]. ^2^Expression of MtCK and CKB was calculated from immunohistochemistry. ^3^Statistical difference was calculated by Student - *t* test. Mean ± SD. ^4^tatistical difference was calculated by paired Student - *t* test. Mean ± SD. Abbreviations: pap: papillary adenocarcinoma; tub1: well-differentiated adenocarcinoma; tub2: moderately differentiated adenocarcinoma; por1: poorly differentiated adenocarcinoma scattered type; por2: poorly differentiated adenocarcinoma solid type; muc: mucinous adenocarcinoma; sig: signet ring cell carcinoma; pT1-pT2: carcinoma invading within muscularis propria layer; pT3-pT4: carcinoma invading subserosal layer or serosa; pN0: no nodal metastasis; pN1-pN2: nodal metastases in regional lymph nodes; pM0: no distant metastasis; pM1: distant metastasis positive.

### Inhibitory effect of DNFB on CKB and MTCK

**Figure 2:**
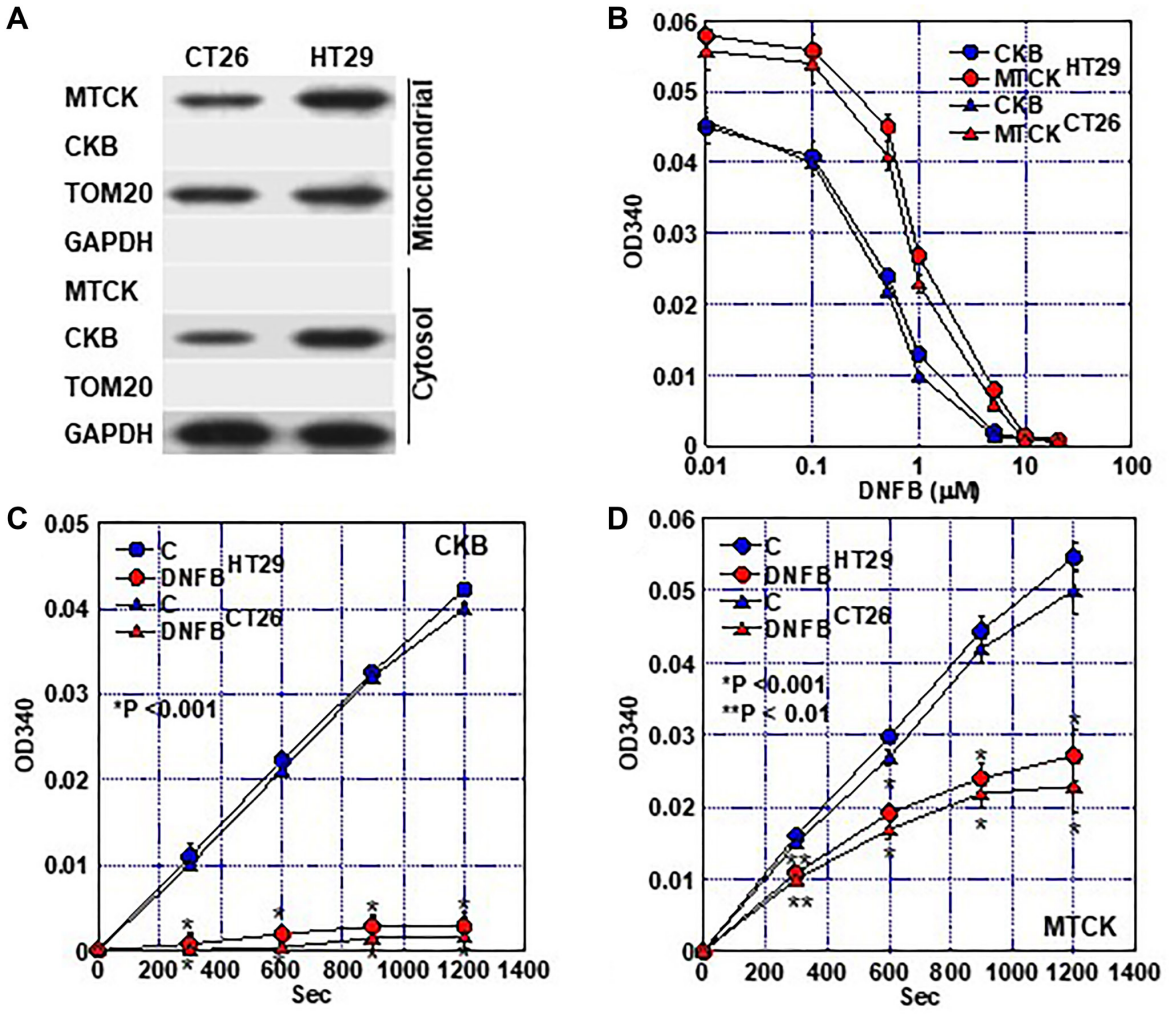
Creatine kinase inhibitory effect of DNFB. (**A**) Protein fractionation of mitochondria (Mitochondrial) and mitochondria-free cytosol (Cytosol). MTCK and CKB were detected by western blotting. TOM20 and GAPDH were subjected as a mitochondrial marker and a cytosol marker, respectively. (**B**) Inhibition of creatine kinase activity (at 20 min) of CKB (cytosol fraction) and MTCK (mitochondrial fraction) in a DNFB concentration-dependent manner. (**C**, **D**) Inhibition of creatine kinase activity of CKB (C) and MTCK (D) in a time-dependent manner. Error bar, standard deviation of three independent trials. Statistical significance was calculated using a two-tailed ordinary analysis of variance. Abbreviations: CKB: creatine kinase B; MTCK: mitochondrial creatine kinase; DNFB: dinitrofluorobenzene; TOM20: translocase of the outer membrane 20; GAPDH: glyceraldehyde 3-phosphate dehydrogenase.

In this study, dinitrofluorobenzene (DNFB) was used as an inhibitor of CK activity and its effect was examined in CT26 and HT29 colon cancer cells (Figure 2). Proteins were extracted from both cells and the mitochondrial and cytoplasmic fractions were separated. In both fractions, protein levels of CKB and MTCK were examined. HT29 cells showed higher levels of CKB and MTCK than those in CT26 cells (Figure 2A). In HT29 cells, DNFB decreased the CK activity in a concentration-dependent manner, with IC50 of 0.87 μM for MTCK and 0.63 μM for CKB (Figure 2B). In CT26 cells, DNFB also showed dose-dependent inhibition of MTCK (IC50 = 0.84 μM) and CKB (IC50 = 0.60 μM). Treatment with 2 μM DNFB reduced the activities of CKB and MTCK in a time-dependent manner and decreased the CK activities to 6% and 48%, respectively, after 20 min in HT29 cells (Figure 2C, 2D). In CT26 cells, DNFB decreased activities of CKB and MTCK to 5% and 46%, respectively.

### Effect of creatine shuttle inhibition on CRC cells

**Figure 3:**
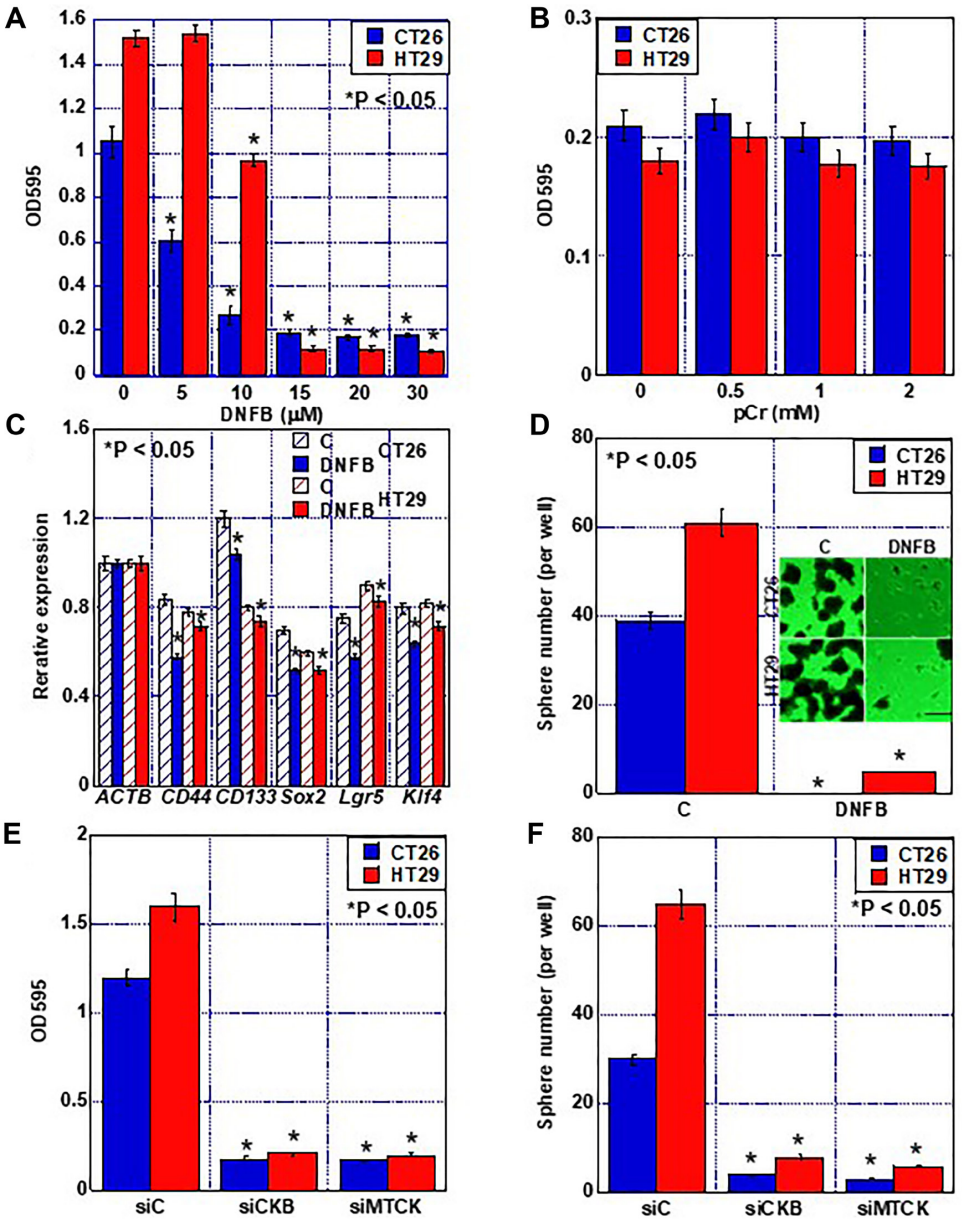
Effect of creatine shuttle inhibition by DNFB on growth and stemness of CRC cells. (**A**) Effect of DNFB on cell growth. (**B**) Effect of pCr on DNFB-treated CRC cells. (**C**) Effect of DNFB (10 μM) on expression of stemness-associated genes detected by quantitative RT-PCR. β-Actin was used as a loading control. (**D**) Effect of DNFB (10 μM) on sphere formation. Scale bar, 50 μm. (**E**, **F**) Effect of knockdown of CKB or MTCK on cell proliferation (E) and sphere formation (F). Error bars: standard deviation of three independent trials. Statistical significance was calculated using a two-tailed ordinary analysis of variance. Abbreviations: CKB: creatine kinase B; MTCK: mitochondrial creatine kinase; DNFB: dinitrofluorobenzene; CRC: colorectal cancer; OD: optimal density; pCr: phosphocreatine; C: untreated control.

The effect of creatine shuttle inhibition by DNFB treatment on cell proliferation was examined (Figure 3A). The number of both CT26 and HT29 cells decreased in a DNFB con-centration-dependent manner, with IC50 values of 6.9 μM and 12.3 μM, respectively. HT29 cells are less sensitive to DNFB than CT26 cells, possibly related to higher protein expression of CKB and MTCK. Next, the rescue effect of phosphocreatine on growth inhibition by DNFB was examined; however, no rescue was observed at any concentration (Figure 3B). Investigation of the effect of creatine shuttle suppression on stem cell marker expression showed that the gene expression of CD44, CD133, SOX2, LGR5, and KLF4 was reduced in both CT26 and HT29 cells (Figure 3C). Furthermore, when the sphere formation ability was examined, the number of spheres was significantly reduced by the suppression of the creatine shuttle (Figure 3D). To demonstrate that the inhibitory effect of DNFB on cell proliferation and stemness is specific to CK inhibition, we performed knockdown of CKB and MTCK (Figure 3E, 3F), and found that knockdown of CKB or MTCK inhibited proliferation and sphere forming ability comparable to DNFB.

### Effect of inhibition of creatine shuttle on mitochondria

**Figure 4:**
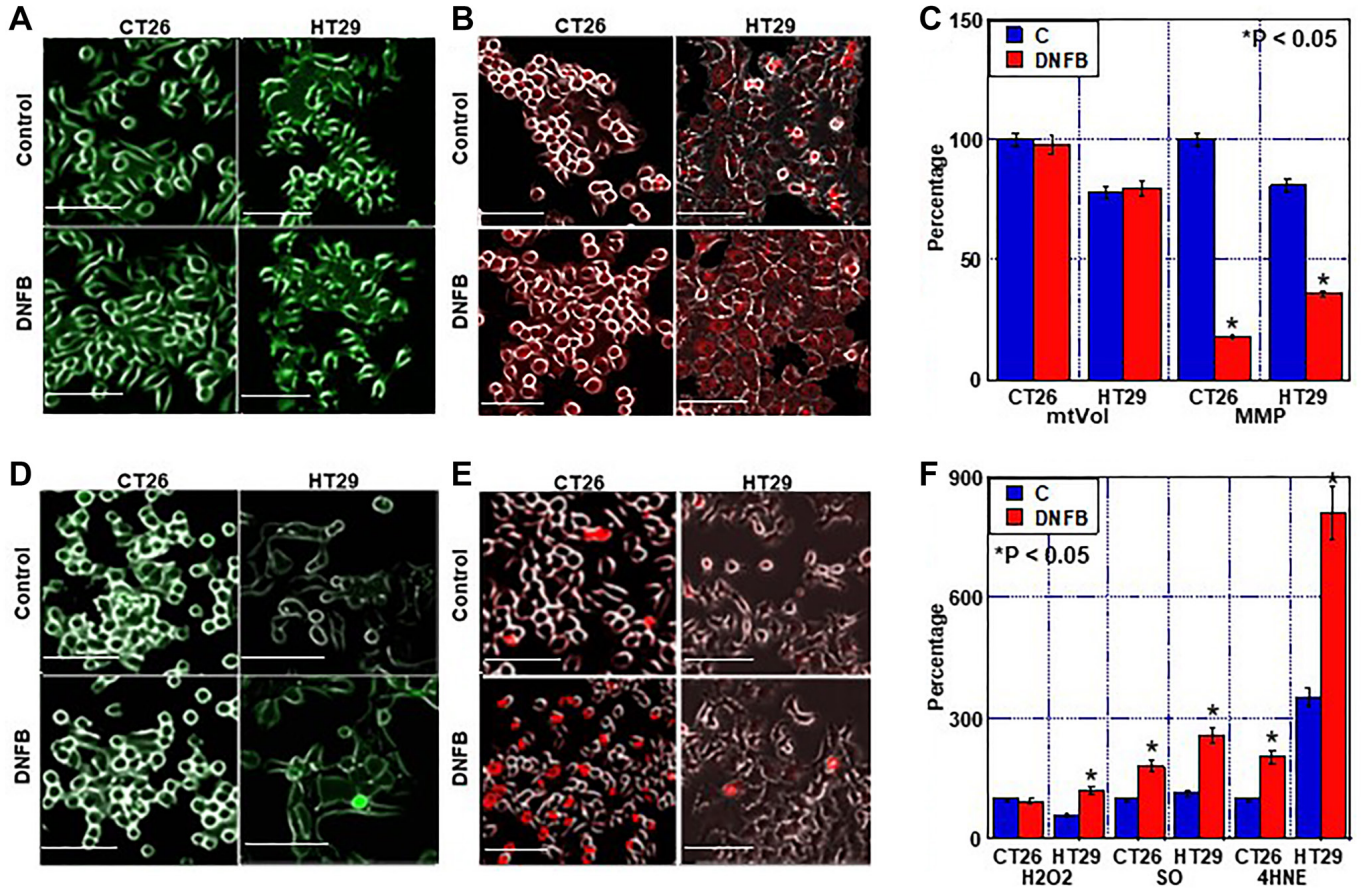
Effect of creatine shuttle inhibition by DNFB on mitochondrial function in CRC cells. (**A**–**C**) Effect of DNFB (5 μM) on mitochondrial volume (mtVol) (A) and mitochondrial membrane potential (MMP) (B), and their semi-quantification (C). (**D**–**F**) Effect of DNFB on mitochondrial ROS production. (D) H2O2, (E) SO and (F) semi-quantification of H2O2, SO and 4-HNE. Error bars: standard deviation of three independent trials. Statistical significance was calculated using a two-tailed ordinary analysis of variance. Abbreviations: DNFB: dinitrofluorobenzene; CRC: colorectal cancer; SO: superoxide; HNE: hydroxynonenal.

**Figure 5:**
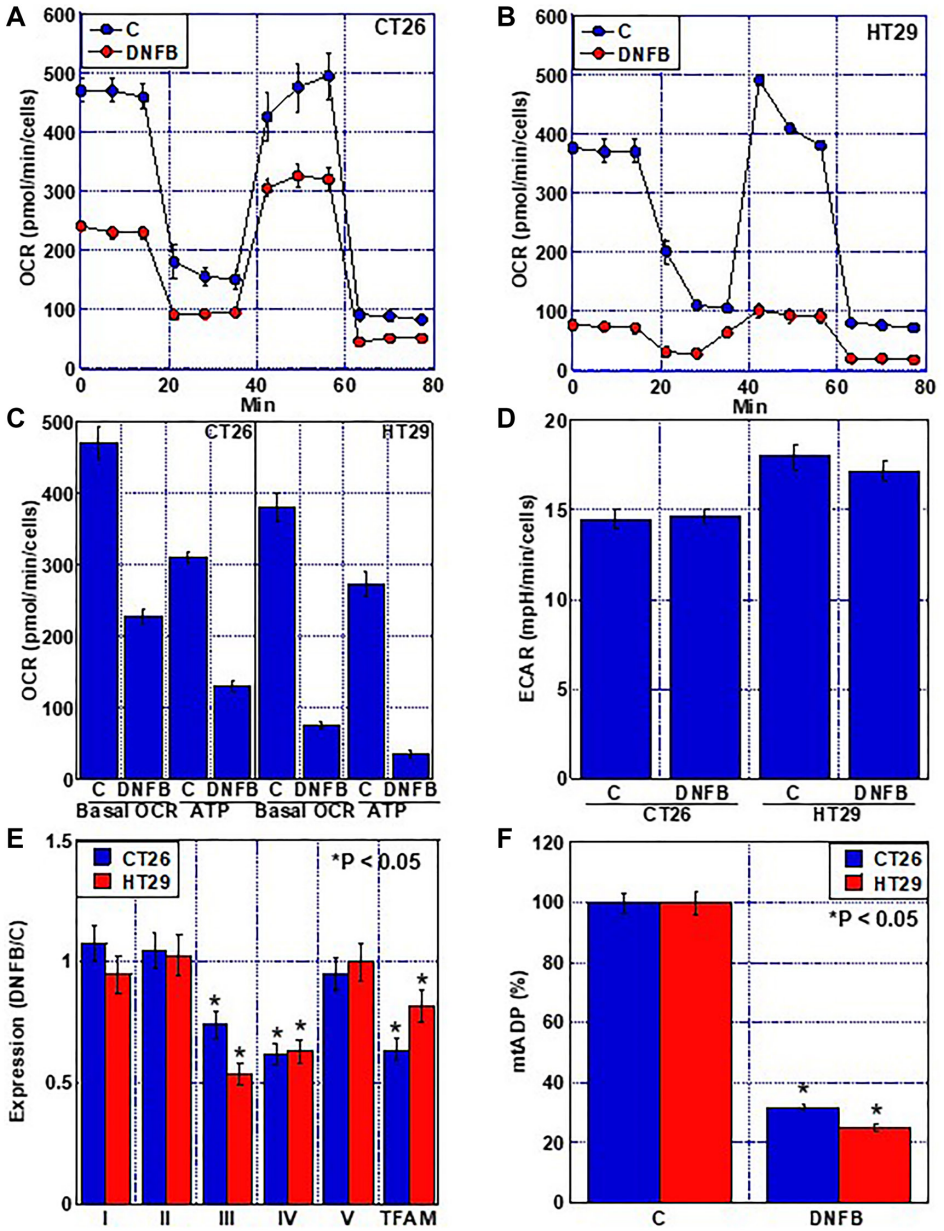
Effect of creatine shuttle inhibition by DNFB on energy metabolism in CRC cells. (**A**, **B**) Effect of DNFB (5 μM) on mitochondrial respiration flux analysis. (**C**, **D**) Effect of DNFB on basal OCR, ATP production (C) and ECAR (D). (**E**) Expression of each ETC complex indicated as a ratio of the expression in DNFB-treated cells to that in untreated control cells (DNFB/C). (**F**) ADP con-centration in the mitochondrial fraction. Error bars: standard deviation of three independent trials. Statistical significance was calculated using a two-tailed ordinary analysis of variance. Abbreviations: DNFB: dinitrofluorobenzene; CRC: colorectal cancer; OCR: oxygen consumption rate; ECAR: extracellular acidification rate; ETC: electron transfer chain.

Next, we examined the effect of creatine shuttle inhibition by DNFB on mitochondria (Figures 4 and 5). Inhibition of the creatine shuttle did not alter the mitochondrial volume (Figure 4A, 4C). In contrast, inhibition of the creatine shuttle decreased the mitochondrial membrane potential in both cell lines (Figure 4B, 4C). When mitochondrial reactive oxygen species (ROS) was examined, inhibition of the creatine shuttle increased superoxide and lipid peroxide (4-HNE) in CT 26 cells (Figure 4D–4F). In contrast, inhibition of the creatine shuttle increased H2O2, superoxide, and 4-HNE in HT29 cells.

Examination of mitochondrial respiration indicated that inhibition of the creatine shuttle in both cell types decreased the oxygen consumption rate (OCR) and ATP production (Figure 5A–5C). In contrast, the extracellular acidification rate (ECAR), an indicator of glycolysis, remained unchanged (Figure 5D). Investigation of the expression of mitochondrial DNA-encoded genes in each complex of the electron transport system and mitochondrial transcription factor A (TFAM) revealed that the expression levels of complexes III and IV and TFAM were reduced by inhibition of the creatine shuttle (Figure 5E). Intramitochondrial ADP was decreased by inhibition of the creatine shuttle (Figure 5F).

### Effect of creatine shuttle inhibition on cancer metastasis

**Figure 6:**
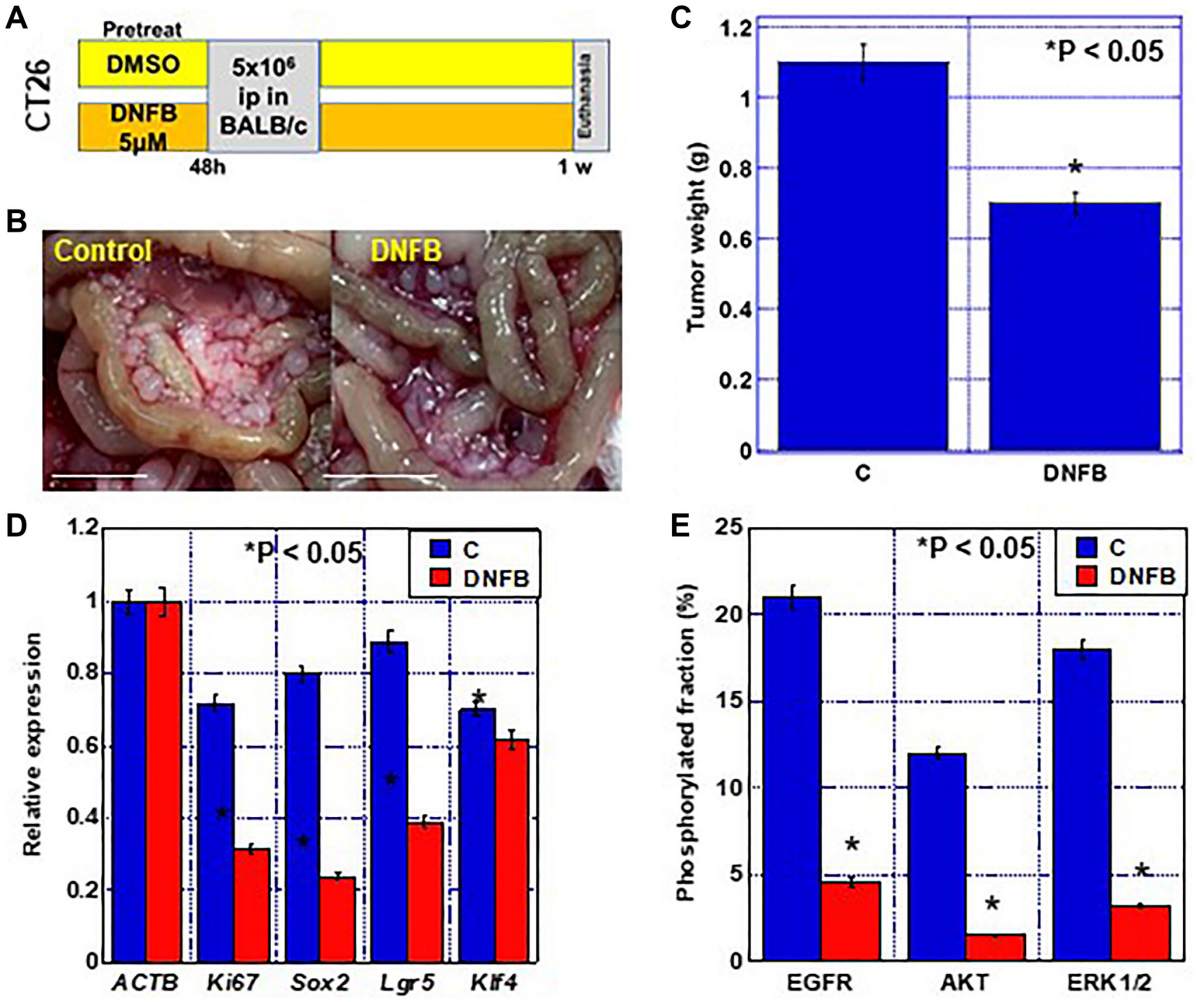
Effect of creatine shuttle inhibition by DNFB on peritoneal dissemination of CT26 cells. (**A**) Experimental protocol. CT26 cells (5 × 10^**6**^) pretreated with DNFB (5 μM) or DMSO (vehicle) were inoculated into the peritoneal cavity of each mouse (*n* = 5). (**B**) Macroscopical appearance of peritoneal tumors. Scale bar, 5 mm. (**C**) Wight of the peritoneal tumors. (**D**) Expression of stem-ness-associated genes by quantitative RT-PCR. (**E**) Effect of DNFB on phosphorylation of EGFR, AKT, and ERK1/2 by ELISA. Phosphorylation fraction = phosphorylated protein/total protein (%). Error bars: standard deviation in five mice or three independent trials. Statistical significance was calculated using a two-tailed ordinary analysis of variance. Abbreviations: DNFB: dinitrofluorobenzene; EGFR: epithelial growth factor receptor; pEGFR: phosphorylated EGFR; pAKT: phosphorylated AKT; ERK: extracellular signal-regulated kinase; pERK1/2: phosphorylated ERK1/2; ELISA: enzyme-linked Immunosorbent Assay.

The effect of creatine shuttle inhibition on cancer metastasis was examined using a model in which CT26 cells were disseminated into the peritoneal cavity of syngeneic BALB/c mice (Figure 6). Since DNFB is adsorbed onto plasma proteins such as albumin and the blood concentration is not maintained [15], CT26 cells were pretreated with DNFB and then inoculated (Figure 6A). Tumors formed in the peritoneal cavity reduced by 62% after DNFB treatment (Figure 6B, 6C). The expression of Ki-67, a proliferation marker, and of Sox2, Lgr5, and Klf4, which are stem cell markers, was decreased in the excised tumors (Figure 6D). Furthermore, when phosphorylation of major tumor-promoting phosphorylation signals was examined, examined, the phosphorylated levels of EGFR, AKT, and ERK1/2 were markedly reduced (Figure 6E).

### Effect of creatine shuttle inhibition on phosphate signaling

**Figure 7:**
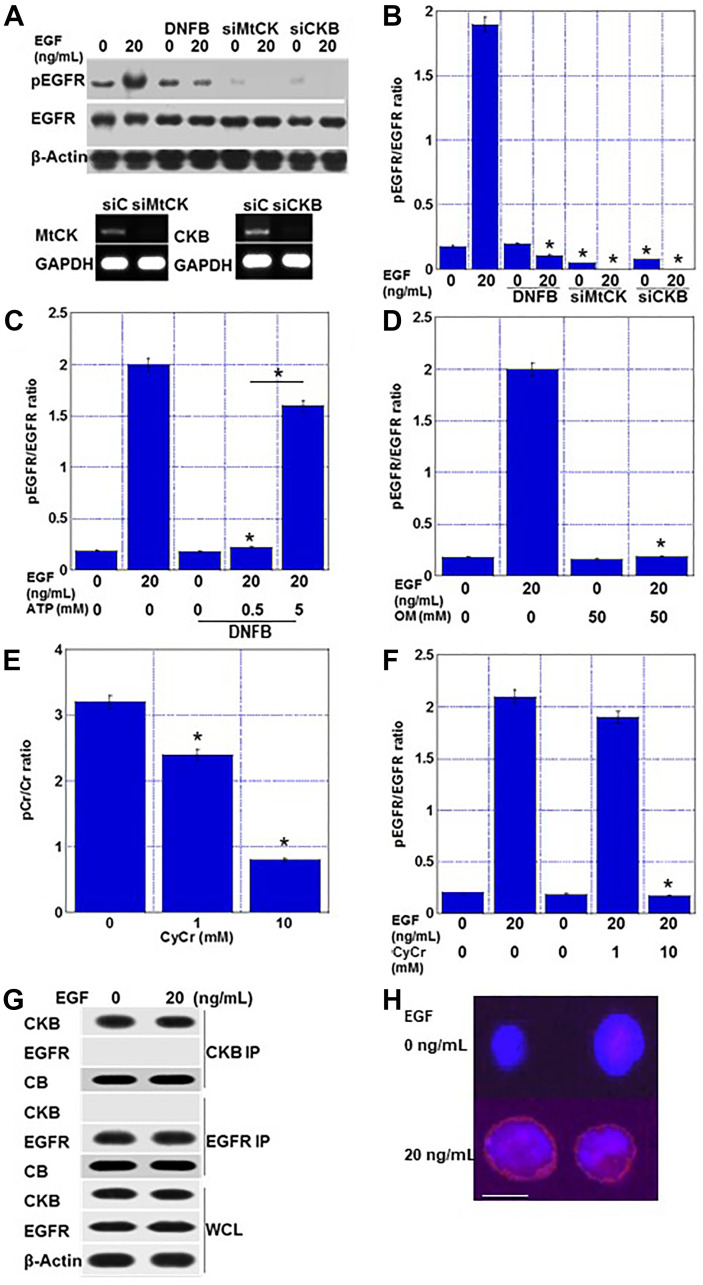
Creatine shuttle provides ATP for EGFR phosphorylation in EGF-treated HT29 cells. (**A**) Effect of DNFB (5 μM) and knockdown of MTCK or CKB on EGFR phosphorylation. Lower panels, effect of siMTCK and siCKB on expression of MTCK and CKB, respectively. (**B**) Semi-quantification of EGFR phosphorylation levels. (**C**) Effect of ATP on suppressed EGFR phosphorylation by DNFB (5 μM). (**D**) Effect of oligomycin on EGFR phosphorylation. (**E**) Effect of CyCr on creatine phosphorylation. (**F**) Effect of CyCr on EGFR phosphorylation. (**G**) Co-immunoprecipitation using anti-CKB antibody or anti-EGFR antibody to examine binding of CKB and EGFR. (**H**) Duolink^®^ proximity ligation assay. Red, proximity signal of EGFR and CKB. Blue, DAPI. Scale bar, 10 μm. Error bars: standard deviation of three independent trials. Statistical significance was calculated using a two-tailed ordinary analysis of variance. Abbreviations: DNFB: dinitrofluorobenzene; EGFR: epithelial growth factor receptor; pEGFR: phosphorylated EGFR; EGF: epithelial growth factor; CKB: creatine kinase B; MTCK: mitochondrial creatine kinase; siC: short interfering RNA (siRNA) used as control; siMTCK: siRNA for MTCK; siCKB: siRNA for CKB; GAPDH: glyceraldehyde 3-phosphate dehydrogenase; OM: oligomycin; CyCr: cyclocreatine; pCr: phosphocreatine; IP: immunoprecipitation; CB: Coomassie blue; WCL: whole cell lysate; DAPI: 4′,6-diamidino-2-phenylindole.

We hypothesized that the creatine cycle supplies ATP to the phosphorylation signal because animal experiments have shown that CRC cells treated with DNFB exhibit a wide range of phosphorylation signal suppression (Figure 7). Treatment of HT29 cells with epithelial growth factor (EGF) increased the EGFR phosphorylation levels, whereas co-treatment with EGF and DNFB decreased the phosphorylation levels (Figure 7A, 7B). Knockdown of MTCK or CKB in HT29 cells resulted in decreased EGFR phosphorylation in the absence of EGF treatment and almost no phosphorylation after EGF treatment. Furthermore, DNFB-induced EGFR phosphorylation inhibition was not rescued by ATP administration at 0.5 mM, but was rescued at a high concentration of 5 mM (Figure 7C). EGFR phosphorylation was inhibited when ATP production in the electron transport chain was inhibited by oligomycin (Figure 7D). Cyclocreatine, an inactive derivative of creatine, inhibited phosphocreatine production (Figure 7E). Treatment with cyclocreatine inhibited EGFR phosphorylation (Figure 7F). Together, these results suggested that the creatine cycle is an efficient ATP donor for phosphorylation.

Finally, we examined whether CKB and EGFR were physically associated with each other. Immunoprecipitation was used to examine whether CKB and EGFR exhibited molecular binding, but no binding was observed, regardless of the presence or absence of EGF treatment (Figure 7G). However, the DuoLink proximity ligation assay indicated that CKB and EGFR were in close proximity as a signal was generated after EGF treatment (Figure 7H).

## DISCUSSION

In this study, we showed that inhibition of the creatine shuttle by blocking CKB and MTCK activity suppressed the growth, stemness, and metastasis of cancer. It was suggested that the cause of this is related to inhibition of both mitochondrial energy metabolism and the phosphorylation signaling system.

In this study, it was suggested that the creatine shuttle via CKB and MTCK may supply ATP for phosphorylation. Phosphorylation signals are essential for cellular activity, but the source of ATP for the phosphorylation process is not specific and is thought to be due to the passive diffusion of ATP within the cytoplasm [16]. This intra-cellular imbalance is thought to cause clustering of molecules and organelles [17].

However, the NLR family pyrin domain containing 3 (NLRP3) inflammasome is supplied with ATP produced in the mitochondria via phosphocreatine [18]. This finding suggests the existence of an active ATP supply in the cytoplasm. Our data show that, similar to the NLRP3 inflammasome, ATP generated by mitochondrial oxidative phosphorylation is translocated to the cytoplasm by the creatine shuttle, and ATP retrieved by CKB is used for EGFR phosphorylation. This indicates that the creatine shuttle overcomes the diffusion limitations of ATP [6]. The role of the creatine shuttle as an ATP donor to phosphorylation signals needs to be confirmed by comprehensive phosphorylation analysis.

Our data did not reveal a regulation of the spatial arrangement of the ATP donor CKB and recipient EGFR. The direct binding of both proteins has not been reported, and we could not confirm this by immunoprecipitation. However, we found that ligand stimulation brought both proteins into close proximity by DuoLink assay. In this assay, fluorescence occurs when two proteins are within 40 nm [19]. This suggested the existence of a mechanism that brings CKB and EGFR spatially close. Some chaperones or the cytoskeleton may be involved [20]; however, further investigation is required.

In this study, inhibition of the creatine shuttle suppressed mitochondrial respiration, decreased mitochondrial membrane potential, and increased mitochondrial ROS levels. This finding indicates that the creatine shuttle is not the only pathway for exporting the energy produced in the mitochondria but also affects various mitochondrial functions. Another significance of the creatine shuttle is that it does not deplete ADP in mitochondria during energy export. A strict ADP gradient exists within the mitochondria and is necessary for ATP production [21]. Another pathway for the export of energy produced in mitochondria is the export of ATP by adenine nucleotide translocator (ANT). In this case, there is a risk of ATP and ADP depletion in the mitochondria, which reduces ATP production. By contrast, the creatine shuttle retains ADP when MTCK generates phosphocreatine from ATP. The ADP-ATP cycle in the mitochondria is thought to be maintained in the creatine shuttle. This suggests that suppression of the creatine shuttle by inhibition of CK activity results in decreased mitochondrial respiration owing to ADP depletion. It has also been reported that destabilization of the creatine shuttle is linked to mitochondrial DNA disorders, suggesting that the creatine shuttle may be involved in mitochondrial stabilization [22]. Inhibition of CK induces mitochondrial inner membrane damage [23], suggesting that CK activity is required for the maintenance of mitochondria.

Reduced mitochondrial respiration due to creatine shuttle inhibition can be compensated in cancer cells by glycolysis and lactate fermentation, known as the Warburg effect. However, our data did not reveal an increase in glycolysis. It has been reported that intracellular ATP cannot be maintained by cyclocreatine treatment [24]. In addition, it has been suggested that ATP turnover in glycolysis, oxidative phosphorylation, and the creatine shuttle may exhibit parallel kinetics [25]. Impairments in the energy metabolism may not be replaced by other systems.

Another unique finding of this study was that the creatine shuttle is closely correlated with cancer stemness. Creatine shuttles have attracted attention for their importance in energy metabolism, but their relationship with stemness has not been clarified. Recently, stem cells have been shown to produce energy through oxidative phosphorylation, revealing a relationship between stemness and oxidative phosphorylation [4]. Low levels of ROS are required for stem cell maintenance in iPS cells [26]. In contrast, inhibition of the creatine shuttle inhibits oxidative phosphorylation and increases mitochondrial ROS production, impairing mitochondrial stem cell maintenance. However, it is not clear from this study whether stemness is specific to the creatine shuttle and further investigation is required.

In our study, CKB and MTCK were upregulated in CRC. Although little is known about the regulation of CKB and MTCK expression, the most important point is that CKB expression is suppressed by p53 and enhanced by p53 silencing [27, 28]. The p53 mutation, which is frequently found in CRC, is thought to lead to the upregulation of CKB. Furthermore, since MTCK expression changes in concert with CKB expression [29], it is thought that the expression of both is upregulated in CRC. In contrast, there are no reports regarding the involvement of microsatellite instability in the expression of CKB and MTCK. We have shown that the creatine shuttle plays an important role in energy metabolism and phosphorylation signaling in CRC. In contrast, MTCK expression is decreased in high-grade prostate cancer [30]. The possibility that there is an ATP donor that replaces the creatine shuttle cannot be denied.

In this study, DNFB was used as an inhibitor of CK activity. DNFB is known as a skin sensitizer [31] that enhances inflammation-induced skin tumorigenicity [32]. In contrast, DNFB has a specific inhibitory effect on creatine kinase [33] and induces mitochondrial inner membrane damage [23]. We showed that DNFB inhibited both CKB and MTCK; however, the inhibitory effect was stronger with CKB. This may be due to difference of affinity of DNFB. At the cellular level, DNFB delivery is further involved: narrowing of drug delivery to the extracellular, endosomal, cytoplasmic, and mitochondrial areas results in progressively lower drug concentrations [34].

Because DNFB reacts rapidly with amino groups to produce 2,4-dinitrophenylamine, it binds to plasma albumin and α1-acid glycoprotein, resulting in phenylation, and is not delivered to target tissues [15]. For this reason, it is difficult to adapt DNFB to the living body. Cyclocreatine is phosphorylated by CK, but does not act as a phosphate donor. It inhibits phosphocreatine production through competitive inhibition with creatine; however, its effect is milder than that of DNFB. Therefore, even 5 mM cyclocreatine did not show strong growth inhibition *in vitro* [35]. Our results showed that 10 mM cyclocreatine inhibited EGFR phosphorylation. Our data suggest that the antitumor effect of creatine shuttle inhibition can be attributed to the inhibition of mitochondrial energy production as well as the inhibition of multiple phosphorylation signals through inhibition of the ATP supply. Therefore, it is necessary to develop a new CK inhibitor to induce these two effects *in vivo*.

## MATERIALS AND METHODS

### Tissue microarray

A human colorectal adenocarcinoma tissue microarray (product ID: BC000110 and BC051111), which contained 184 cases of CRCs, was purchased from US Biomax, Inc. (Rockville, MD, USA). Clinicopathological parameters were obtained from the data pro-vided by the provider. All procedures were performed in accordance with the Ethical Guidelines for Human Genome/Gene Research issued by the Japanese Government and were approved by the Ethics Committee of Nara Medical University (Approval Number 937, 2018/4/1).

### Immunohistochemistry

Tissue microarray slides were processed for the immunohistochemical analysis of CKB and MTCK. Tissue micro array slides were incubated with antibodies against CKB (0.2 μg/mL, ab151579, Abcam, Cambridge, MA, USA) and MTCK1 (0.2 μg/mL, 89-7263-83, Abbexa Ltd., Cambridge, UK) and appropriate secondary antibodies (Medical and Biological Laboratories, Nagoya, Japan) (all 0.2 μg/mL). The tissue sections were then color-developed with diamine benzidine hydrochloride (Dako, Glostrup, Denmark) and counterstained with Meyer’s hematoxylin (Sigma-Aldrich Chemical Co., St. Louis, MO, USA). To evaluate CKB and MTCK, we counted cells that exhibited immunoreactivity in the cytoplasm and scored staining strength as grade 0–3. The product of the grade (0–3) and frequency of positive cells (0–1) is the expression index.

### Cell lines and reagents

The HT29 human carcinoma cell line was purchased from Dainihon Pharmacy Co. (Tokyo, Japan). The CT26 murine colon carcinoma cell line was a gift from Professor I. J. Fidler (MD Anderson Cancer Center, TX, USA). Cells were cultured in Dulbecco’s modified Eagle’s medium supplemented with 10% fetal bovine serum at 37°C in 5% CO_2_.

1-Fluoro-2,4-dinitrobenzene (DNFB), human recombinant EGF, mouse recombinant EGF, phosphocreatine (pCr), oligomycin (Sigma), ATP, and cyclocreatine (Wako Pure Chemical Corp. Ltd., Osaka, Japan) were purchased from the indicated manufacturers.

### MTS [3-(4,5-dimethylthiazol-2-yl)-5-(3-carboxymethoxyphenyl)-2-(4-sulfophenyl) -2H-tetrazolium] assay

MTS assays were performed using the CellTiter 96 Aqueous One Solution Cell Proliferation Assay kit (Promega Biosciences, Inc., San Louis, MO, USA). The plates were read on a multiscan FC microplate photometer at a wavelength of 490 nm. The MTS value in cells cultured with the control oligonucleotide was used as the control.

### Small interfering RNA

Stealth Select RNAi (siRNA) targeting human CKB, human MTCK, mouse CKB and mouse MTCK was purchased from Sigma. AllStars Negative Control siRNA was used as the control (Qiagen, Valencia, USA). The cells were transfected with 10 nM siRNA using Lipofectamine 3000 (Thermo Fisher Scientific) according to the manufacturer’s recommendations.

### Mitochondrial imaging

The mitochondrial function was examined using fluorescent probes. Cells were incubated with the probes for 30 min at 37°C and then imaged using a BZ-X710 all-in-one fluorescence microscope (KEYENCE, Osaka, Japan). We used MitoROS to detect superoxide (10 μM, AAT Bioquest Inc., Sunnyvale, CA, USA) and dihydrorhodamine 123 to detect H2O2 (10 μM, Sigma-Aldrich) to assess oxidative stress, MitoGreen to detect mitochondrial volume (100 nM, PromoCell GmbH, Heidelberg, Germany), and tetrathylrhodamine ethyl ester (200 nM, Sigma-Aldrich) to assess mitochondrial membrane potential.

### Extracellular flux analysis

To analyze mitochondrial respiration and ATP production, we used a Seahorse XF Analyzer (Agilent Technologies, Santa Clara, CA, USA) to measure extracellular flux in live cells. The cells were collected immediately after treatment, transferred to the wells of an XF plate at a density of 2 × 10^4^ cells/well, and incubated overnight. The following day, the medium in the XF plate was replaced with XF DMEM 1 h prior to the assay, and a Mito Stress Test (Seahorse XF Cell Mito Stress Test, Agilent) was performed according to the manufacturer’s protocol. The OCR was measured under the following conditions: 2 μM oligomycin, 0.5 μM carbonyl cyanide-p-trifluoromethoxyphenylhydrazone, and 0.5 μM rotenone/antimycin A. OCR was normalized to the total cellular protein concentration, which was determined after protein extraction from the analyzed cells.

### Protein extraction

To prepare whole-cell lysates, cells were washed twice with cold PBS, harvested, and lysed with RIPA buffer containing 0.1% sodium dodecyl sulfate (SDS) (Thermo Fisher Scientific, Tokyo, Japan) [36]. Cell fractions were extracted by processing the cells with a Cell Fractionation Kit (Abcam), according to the manufacturer’s instructions [37]. Protein assays were performed using the Protein Assay Rapid Kit (Wako).

### Immunoblot analysis

Whole cell lysates were prepared as previously described [38]. Lysates (50 μg) were subjected to immunoblot analysis on 12.5% SDS-polyacrylamide gels, followed by electrotransfer onto nitrocellulose membranes (Bio-Rad, Hercules, CA, USA). Membranes were incubated with primary antibodies and then with peroxidase-conjugated IgG secondary antibodies (MBL, Nagoya, Japan). Primary antibodies against EGFR, ERK1/2 (Santa Cruz Biotechnology Inc., Santa Cruz, CA, USA), phosphorylated EGF receptor (pEGFR Tyr1068) (Cell Signaling Technology Japan, Tokyo, Japan) were used. Antibodies against β-actin (Oncogene Research Products, Cambridge, MA, USA) were used to assess the protein loading. Immune complex binding was visualized using a CSA system (DAKO, Carpinteria, CA, USA).

### Immunoprecipitation

Immunoprecipitation was performed as previously described [39]. Lysates were pre-cleaned in lysis buffer containing protein A/G agarose (Santa Cruz) for 1 h at 4°C and subsequently centrifuged. The supernatants were then incubated with a precipitation antibody against EGFR (Santa Cruz) or CKB (Boster Immunoleader, Pleasanton, CA, USA) and protein A/G agarose for 1.5 h at 4°C. Precipitates were collected by centrifugation, washed three times with wash buffer, and solubilized with 4× Laemmli sample buffer (Bio-Rad, Hercules, CA, USA) and 2-mercaptoethanol (Sigma). Immunoblotting was performed using antibodies against EGFR (Santa Cruz Biotechnology) or CKB (Boster).

### Enzyme-linked immunosorbent assay (ELISA) and activity assay

An ELISA kit was used to measure the concentrations of 4-hydroxynonenal (4-HNE, Cusabio Technology, Houston, TX, USA), pEGFR (RayBiotech Life, Peachtree Corners, GA, USA), EGFR, pAKT, AKT (Abcam), pERK1/2, ERK (Enzo Life Science, Farmingdale, NY, USA), pCr (ELK Biotechnology, Hubei, China), creatine (Cr, Abcam), and ADP (LSBoi, Seattle WA, USA). CK activity was measured using a Creatine Kinase Assay Kit (Abnova, Taipei, Taiwan). The assay was performed using whole-cell lysates according to the manufacturer’s instructions.

### Reverse transcription-polymerase chain reaction (RT-PCR)

To assess mRNA expression, RT-PCR was performed with 2 μg of total RNA extracted from CT26 and HT29 cells using TRI REAGENT (Molecular Research Center, Inc., Cincinnati, OH, USA) according to the manufacturer’s protocol. cDNA was synthesized with 0.5 μg total RNA using the Prime Script RT reagent kit together with gDNA Eraser (Perfect Real Time; Takara, Kyoto, Japan) in accordance with the manufacturer’s instructions. Gene expression was analyzed using qRT-PCR, with reactions performed in triplicate using a SYBR Green PCR kit (Takara). Primer sets used in this study are listed in Table 2. Primers were synthesized by Sigma Genosys (Ishikari, Japan). The PCR products were electrophoresed on a 2% agarose gel and stained with ethidium bromide. β-actin mRNA was also amplified for use as an internal control.

**Table 2:** Primer sets

Gene	Animal	Gene ID		Sequence
*CD44*	Human	FJ216964.1	Forward	AAGGTGGAGCAAACACAACC
			Reverse	AGCTTTTTCTTCTGCCCACA
*cd44*	Mouse	M27130.1	Forward	TGGATCCGAATTAGCTGGAC
			Reverse	AGCTTTTTCTTCTGCCCACA
*CD133*	Human	BC012089.1	Forward	TTGTGGCAAATCACCAGGTA
			Reverse	TCAGATCTGTGAACGCCTTG
*cd133*	Mouse	BC028286.1	Forward	GAAAAGTTGCTCTGCGAACC
			Reverse	TCTCAAGCTGAAAAGCAGCA
*SOX2*	Human	NM_003106.4	Forward	AACCCCAAGATGCACAACTC
			Reverse	CGGGGCCGGTATTTATAATC
*sox2*	Mouse	NM_011443.4	Forward	CACAACTCGGAGATCAGCAA
			Reverse	CTCCGGGAAGCGTGTACTTA
*LGR5*	Human	AF061444.1	Forward	CTCTTCCTCAAACCGTCTGC
			Reverse	GATCGGAGGCTAAGCAACTG
*lgr5*	Mouse	NM_010195.2	Forward	CATTCACTTTTGGCCGTTTT
			Reverse	AGGGCCAACAGGACACATAG
*KLF4*	Human	KJ901962.1	Forward	CCCACACAGGTGAGAAACCT
			Reverse	ATGTGTAAGGCGAGGTGGTC
*klf4*	Mouse	NM_010637.3	Forward	CTGAACAGCAGGGACTGTCA
			Reverse	GTGTGGGTGGCTGTTCTTTT
*Ki-67*	Mouse	X82786.1	Forward	GACAGCTTCCAAAGCTCACC
			Reverse	TGTGTCCTTAGCTGCCTCCT
*C-I*	Human	JN034131.1	Forward	CCTGACTCCTACCCCTCACA
			Reverse	ATCGGGTGATGATAGCCAAG
*c-I*	Mouse	NM_025358.3	Forward	ACACAGACCTGGTGGAGACC
			Reverse	GGATGGGCTTGGAGTAATCA
*C-II*	Human	KR710499.1	Forward	TCGCACTGTGCATAGAGGAC
			Reverse	ATATATGCCTGTGGGGTGGA
*c-II*	Mouse	NM_025358.3	Forward	ACTGTGTTTGGGGCTACAGG
			Reverse	GATTGATGACCACGTTGCTG
*C-III*	Human	NM_003366.4	Forward	ATGGCTTTGATTGGACTTGG
			Reverse	CAAAAGCAGCATGGACAAGA
*c-III*	Mouse	NM_025899.2	Forward	GTCAGAGGGCTTCCTGAGTG
			Reverse	ACTCGTCGAGAAAAGGCGTA
*C-IV*	Human	DN994680.1	Forward	TTCATGATCACGCCCTCATA
			Reverse	TAAAGGATGCGTAGGGATGG
*c-IV*	Mouse	AB284309.1	Forward	GGCAGAACGACTCGGTTATC
			Reverse	ACGAAATCAACAACCCCGTA
*C-V*	Human	CR542155.1	Forward	GCGGGACTTCAGTCCTAGTG
			Reverse	CTCGTGCTTGAGATGCTTGT
*c-V*	Mouse	NM_020582.2	Forward	CCTTCCACCGGGATTTTTAT
			Reverse	AATTTGGCAGCTATGGGAGA
*ACTB*	Human	NM_001101.3	Forward	GGACTTCGAGCAAGAGATGG
			Reverse	AGCACTGTGTTGGCGTACAG
*actb*	Mouse	NM_007393.5	Forward	AGCCATGTACGTAGCCATCC
			Reverse	CTCTCAGCTGTGGTGGTGAA

Abbreviations: SOX: SRY-related HMG-box; LGR: leucine-rich repeat-containing G-protein-coupled receptor; KLF: Kruppel-like factor. Electron transfer chain complex: C-I, complex I, NADH dehydrogenase subunit 4 (ND4); C-II, complex II, succinate dehydrogenase complex, subunit A (Sdha); C-III, complex III, ubiquinol cytochrome c reductase core protein 2 (Uqcrc2); C-IV, complex IV, cytochrome c oxidase II (mtCo2); C-V, complex V, ATP synthase, H+ transporting, mitochondrial F0 complex, subunit F2 (Atp5j2).

### Animals

Five-week old male BALB/c mice were purchased from SLC Japan (Shizuoka, Japan). The animals were maintained in a pathogen-free animal facility under a 12/12 h light/dark cycle in a temperature (22°C)- and humidity-controlled environment, in accordance with the institutional guidelines approved by the Committee for Animal Experimentation of Nara Medical University, Kashihara, Japan, following current regulations and standards of the Japanese Ministry of Health, Labor and Welfare (approval nos. 13093, 6/30/2021). Animals were acclimated to their housing for seven days before the start of the experiment. Mice were fed with CE-2 diet (CLEA Japan, Inc., Tokyo, Japan). CT26 cells (5 × 10^6^) pretreated with DNFB (5 μM) or DMSO (vehicle) were inoculated into the peritoneal cavity of male BALB/c mouse (5 week old). Five mice were used in each group. One week after inoculation, the mice were euthanized and intraperitoneal tumors were observed. To measure tumor weight, mice were euthanized by aortic blood removal under the anesthesia sevoflurane (Maruishi Pharmaceutical Co. Ltd., Osaka, Japan) and the peritoneal tumors were dissected from the intestine, mesenterium, diaphragm, and abdominal wall, grossly removing non-tumoral tissues.

### Duolink^®^ proximity ligation assay

The assay was performed following the manufacturer’s instructions. The following is a brief description: HT29 cells (1 × 10^5^) were seeded and incubated for 24 h on Nunc chamber slides (Thermo Fisher), and then incubated with Duolink^®^ Blocking Solution at 37°C for 60 min. Anti-EGFR antibody (mouse monoclonal, 0.5 μg/mL, Santa Cruz) and anti-CKB antibody (rabbit polyclonal, 0.5 μg/mL, Boster) were incubated with the Duolink^®^ Antibody Diluent. The antibody mixture (40 μL) was added to the cell plate after removing Blocking Solution and incubated at 37°C for 2 h. After removing the antibody mixture, MINUS and PLUS probe solutions (each 8 μL) were added and the plate was incubated at 37°C for 1 h. After removing the probe solution and washing, a mixture of ligase and ligation buffer (40 μL) was added and the plate was incubated at 37°C for 30 min. After removing ligation mixture, a mixture of polymerase and amplification buffer (40 μL) was added, followed by incubation at 37°C for 100 min. After removing the probe solutions and washing, the cell plates were mounted with Duolink^®^ *In Situ* Mounting Media containing DAPI. Cells were then observed using a BZ-X710 microscope (KEYENCE, Osaka, Japan).

### Statistical analysis

Statistical significance was calculated using a two-tailed Fisher’s exact test or ordinary analysis of variance (ANOVA) using InStat software (GraphPad, Los Angeles, CA, USA). Correlations were tested using Pearson’s correlation tests. A two-sided *P* value of < 0.05 was considered to indicate statistical significance.

## Abbreviations

CK: creatine kinase
MTCK: mitochondrial creatine kinase
CKB: creatine kinase B
DNFB: dinitrofluorobenzene
CRC: colorectal cancer
EGF: dinitrofluorobenzene
EGFR: epidermal growth factor receptor
ERK: extracellular signal-regulated kinase
ATP: adenosine triphosphate
OCR: oxygen consumption rate
ECAR: extracellular acidification rate
NLRP3: NLR family pyrin domain containing 3
ROS: reactive oxygen species
TFAM: mitochondrial transcription factor A
